# Nutritional Regulation of Muscle Stem Cells in Exercise and Disease: The Role of Protein and Amino Acid Dietary Supplementation

**DOI:** 10.3389/fphys.2022.915390

**Published:** 2022-07-07

**Authors:** Kayleigh M Beaudry, Emileigh R. Binet, Nicolás Collao, Michael De Lisio

**Affiliations:** ^1^ School of Human Kinetics, University of Ottawa, Ottawa, ON, Canada; ^2^ Department of Cellular and Molecular Medicine, Centre for Neuromuscular Disease, University of Ottawa, Ottawa, ON, Canada

**Keywords:** satellite cells, fibro/adipogenic progenitor cells, skeletal muscle, nutrition, leucine

## Abstract

Human skeletal muscle is a remarkedly plastic tissue that has a high capacity to adapt in response to various stimuli. These adaptations are due in part to the function of muscle-resident stem/progenitor cells. Skeletal muscle regeneration and adaptation is facilitated by the activation and expansion of muscle stem cells (MuSCs). MuSC fate is regulated by signals released from cells in their niche, such as fibro-adipogenic progenitors (FAPs), as well as a variety of non-cellular niche components. Sufficient dietary protein consumption is critical for maximizing skeletal muscle adaptation to exercise and maintaining skeletal muscle in disease; however, the role of dietary protein in altering MuSC and FAP responses to exercise in healthy populations and skeletal muscle disease states requires more research. The present review provides an overview of this emerging field and suggestions for future directions. The current literature suggests that in response to resistance exercise, protein supplementation has been shown to increase MuSC content and the MuSC response to acute exercise. Similarly, protein supplementation augments the increase in MuSC content following resistance training. Endurance exercise, conversely, is an area of research that is sparse with respect to the interaction of protein supplementation and exercise on muscle stem/progenitor cell fate. Initial evidence suggests that protein supplementation augments the early myogenic response to acute endurance exercise but does not enhance the MuSC response to endurance training. Resistance training increases the number of proliferating FAPs with no additional effect of protein supplementation. Future research should continue to focus on the nutritional regulation of skeletal muscle stem/progenitor cell fate paired with studies examining the effects of exercise on a variety of human populations.

## Introduction

Skeletal muscle is a remarkably plastic tissue that has a high capacity to adapt in response to different stimuli (Baldwin and Haddad, 2002). In response to resistance exercise, skeletal muscle undergoes hypertrophy and results in an increased muscle mass (Haun et al., 2019). Conversely, muscle disuse and catabolic conditions can cause muscle wasting and degeneration resulting in atrophy (Fanzani et al., 2012). Atrophy can be the result of malnutrition, inactivity, aging, and skeletal muscle disease (Fanzani et al., 2012; Rudrappa et al., 2016). Skeletal muscle mass accounts for roughly 30% of body weight in females and approximately 38% in males (Janssen et al., 2000), and it has an important role in nutrient storage and regulation of metabolism (Wolfe, 2006). Indeed, skeletal muscle contributes significantly to post-prandial glucose disposal, lipid oxidation, resting metabolic rate, and whole-body protein metabolism (Wolfe, 2006; Morton et al., 2015). Further, the role of skeletal muscle as an endocrine organ regulating multiple body systems is emerging (Severinsen and Pedersen, 2020). Therefore, the maintenance and regeneration of muscle mass is critical to ensure health across the lifespan.

Skeletal muscle regeneration is facilitated by the activation and expansion of myogenic muscle stem cells (MuSCs), also known as satellite cells (Wang and Rudnicki, 2011; Wang et al., 2014). MuSCs reside in skeletal muscle, adjacent to myofibers and beneath the basal lamina, in a state of quiescence (Katz, 1961; Mauro, 1961). Muscle damage and signals from cells in their niche, including fibro-adipogenic progenitors (FAPs), cause MuSCs to exit quiescence, proliferate, and differentiate to facilitate muscle repair and adaptation (Feige et al., 2018). To form a mature muscle fibre, MuSCs undergo multiple rounds of proliferation to generate sufficient myonuclei (Bischoff, 1990). Muscle regeneration occurs with several rounds of myoblast fusion and muscle fibre maturation (Knudsen and Horwitz, 1977). Additionally, while the majority of MuSCs go through proliferation and differentiation for skeletal muscle regeneration, a sub-group of MuSCs will undergo cell division to maintain the MuSC pool for future injuries (Collins et al., 2005; Sacco et al., 2008; Dumont et al., 2015). FAPs are muscle resident multipotent stromal cells that are directly involved in muscle adaptation through their promotion of MuSC proliferation and differentiation (Joe et al., 2010; Uezumi et al., 2010; Mozzetta et al., 2013; Uezumi et al., 2021). FAPs have adipogenic, fibrogenic, and osteogenic differentiation potential *in vivo* (Contreras et al., 2021) and are phenotypically identified primarily by their expression of PDGFRα and/or CD90 in humans (Uezumi et al., 2014a; Farup et al., 2021) as well as several recent markers that may distinguish FAP subpopulations (Contreras et al., 2021). FAPs are critical during muscle regeneration to sustain MuSC differentiation and to maintain the MuSC pool during normal development (Wosczyna et al., 2019) and aging (Lukjanenko et al., 2019). However, FAPs have a physiological dichotomy such that in pathological conditions FAP expansion results in an overproduction of fibro/fatty infiltration which leads to impaired myogenesis (Joe et al., 2010; Uezumi et al., 2010; Mozzetta et al., 2013; Contreras et al., 2016; Dong et al., 2017; Gonzalez et al., 2017; Madaro et al., 2018; Stumm et al., 2018).

Adequate dietary protein consumption throughout the lifespan is critical for maintaining optimal skeletal muscle health. Protein consumption plays a substantial role in the attenuation of many skeletal muscle disorders, including those caused by diabetes and age-related loss of muscle mass quantity and function, termed sarcopenia (Beaudry and Devries, 2019). Dietary protein consumption and dietary-derived amino acids have proven to be an effective approach to slow muscle protein catabolism in older adults (Paddon-Jones et al., 2004; Paddon-Jones, 2006). Additionally, protein consumption from a wide variety of sources such as whey supplementation (Park et al., 2019), yogurt (Bridge et al., 2019), whole egg (Van Vliet et al., 2017), and amino acids (Rieu et al., 2006), has a beneficial effect on muscle growth and strength. While a large body of literature and several excellent reviews (Atherton and Smith, 2012; Phillips, 2016; Burd et al., 2019) have described the interaction between dietary protein consumption, exercise, and muscle protein synthesis, the role of dietary protein consumption on MuSC and FAP responses to exercise have been largely unexplored. Therefore, the purpose of the current review is to highlight recent studies examining the role of dietary protein in altering MuSC and FAP responses to exercise, particularly hypertrophy-inducing resistance exercise in healthy populations and skeletal muscle disease states.

### Role of Protein on Skeletal Muscle Mass and the MuSC Niche

Muscle mass is determined, in part, by the relative rates of both muscle protein synthesis (MPS) and muscle protein breakdown (MPB). In the fasted state, rates of MPB exceed MPS which results in a negative protein balance (Biolo et al., 1995). In response to protein consumption and an increase in plasma amino acid availability there will be a subsequent increase in MPS and decrease in MPB which will result in a net positive protein balance (Biolo et al., 1995; Glynn et al., 2010). Exercise also acts synergistically to the effects of protein consumption and augments the muscle protein synthetic response (McLeod et al., 2016; Burd and De Lisio, 2017). This response has been demonstrated following both resistance exercise (Burd et al., 2010; Burd et al., 2011; Reitelseder et al., 2011; Agergaard et al., 2017) and endurance exercise (Pikosky et al., 2006; Harber et al., 2010; Di Donato et al., 2014; Churchward-Venne et al., 2020). This increase anabolic response following exercise is thought to be due to an increased muscle sensitivity to hyperaminoacidemia, resulting in a greater muscle protein synthetic response to lower concentrations of protein intake (Churchward-Venne et al., 2012). In contrast, catabolic conditions such as physical inactivity shifts net protein balance to favour degradation, which, if sustained for an extended period can result in muscle atrophy (Vainshtein and Sandri, 2020). The fine-tune balance between protein synthesis and degradation in skeletal muscle is related to physical activity and nutritional status and can positively or negatively impact the cells of the muscle niche (Atherton and Smith, 2012; Shamim et al., 2018).

Dietary protein consumption is critical for providing the substrates responsible for facilitating skeletal muscle repair and regeneration (Shamim et al., 2018). Amino acids from dietary protein consumption have previously been demonstrated to promote myotube formation *in vitro* and increased *Myod* and *Myogenin* expression in rat skeletal muscle (Dai et al., 2015). Similarly, the leucine metabolite *β*-hydroxy-β-methyl butyrate (HMB) has been demonstrated to enhance proliferation, differentiation, and accelerate fusion in primary myoblasts (Kornasio et al., 2009) and increase MuSC proliferation in neonatal pigs (Kao et al., 2016). These effects may be due to leucine’s role in stimulating mammalian target of rapamycin (mTOR) activity (Sancak et al., 2008). mTOR has long been known for its key role in diverse cellular processes including cell growth, differentiation, and protein synthesis (Laplante and Sabatini, 2012). As such, mTOR is essential for MuSC function and skeletal muscle regeneration through its role in regulating the expression of myogenic genes (Zhang et al., 2015). Rodgers et al. (2014) have previously demonstrated that mTORC1 activity is necessary for MuSCs to transition during quiescence to an alert phase to enhance their regenerative capacity. Additionally, leucine can promote myoblast proliferation and differentiation *in vitro* through an mTORC1-MyoD cascade (Dai et al., 2015). Other essential amino acids (EAA’s) such as methionine has also been identified as a regulator of cell proliferation (Walvekar et al., 2018). Similarly, glutamine is the second most consumed nutrient apart from glucose during the proliferation phase of C2C12 myoblasts (Hosios et al., 2016), suggesting an important role in cell proliferation and its deprivation can lead to an incomplete cell cycle (Gaglio et al., 2009). Thus, *in vitro* and preclinical evidence suggests that dietary amino acids may play a direct role in regulating MuSC fate.

### The Effects of Resistance Exercise and Training With Dietary Protein Manipulation on MuSCs

#### Responses to Acute Resistance Exercise

A large body of evidence has detailed the response of MuSCs after acute resistance exercise in untrained adults. These studies have been excellently reviewed elsewhere (Kadi et al., 2005; Snijders et al., 2015; Murach et al., 2021; Roman and Muñoz-Cánoves, 2022). Resistance exercise is the primary mode of exercise used to elicit positive changes in muscle mass (Schoenfeld et al., 2015) and is composed of contracting the muscles against an external resistance. In general, a single-bout of resistance exercise induces MuSC activation as early as 4-h post-exercise (McKay et al., 2009), with peak activation occurring at 72-h (Bellamy et al., 2014). This has been confirmed in both young and older adults in which increases in MuSC activation were observed between 6 and 24-h post-exercise (Walker et al., 2012; Luk et al., 2019), although the MuSC response in older adults is delayed (Snijders et al., 2014b).

A few recent studies have examined the synergistic effects of protein consumption and acute resistance exercise on MuSC content and activation (Hulmi et al., 2008; Roberts et al., 2010; Farup et al., 2014a; Snijders et al., 2014a; Reidy PT. et al., 2017). Gene expression of the myogenic regulatory factors, *Pax7* and *MyoD*, was similar in whole muscle homogenates between whey protein and placebo groups between 1–6 h after a single bout of resistance exercise in both young (Roberts et al., 2010) and older (Hulmi et al., 2008) adults, suggesting no added benefit of whey protein intake. However, with respect to MuSC content, whey protein intake significantly increased MuSC content in type II fibres 48-h post-unilateral knee extension exercises (Farup et al., 2014a). Type II fibres are commonly referred to as “fast twitch” fibre types and are characterized as having lower oxidative capacity, greater glycolytic capacity, and greater force producing capacity compared to “slow-twitch” type I fibres (Henriksson and Reitman, 1976). Basal concentrations of MuSC’s have been shown to vary between muscle fibre types in healthy and diseased populations (Verdijk et al., 2007; Verney et al., 2008; Verdijk et al., 2009; Snijders et al., 2011; McKay et al., 2012; Suetta et al., 2012; Verdijk et al., 2012; Bankolé et al., 2013; Snijders et al., 2014b) and respond differentially following exercise depending on the type of fibre on which the MuSC is located (McKay et al., 2012; Cermak et al., 2013; Joanisse et al., 2013; Snijders et al., 2014b; Fry et al., 2014). In older adults, ingestion of an EAA supplement resulted in a trend for more Pax7^+^MuSCs 24-h after acute resistance exercise compared to the placebo condition, indicating that there was a trend for an increase in MuSC content (Reidy PT. et al., 2017). Further, MyoD^+^MuSCs and Ki67^+^MuSCs were also significantly increased in the group receiving EAAs versus placebo at 24-h, both indicating that there was an increase in proliferative cells (Reidy PT. et al., 2017). Together, these data suggest that protein supplementation in the form of whey or EAAs augments the MuSC response to acute resistance exercise in both young and older males.

To examine the effects of reduced protein consumption on the MuSC response to acute resistance exercise, Snijders et al. (2014a) restricted dietary protein to 0.1 g/kg/d, which is below the recommended daily allowance (RDA) (0.8 g/kg/d), for 4-days prior to an acute bout of resistance exercise in young males. Interestingly, no differences in MuSC content following resistance exercise were observed in the protein restricted group compared to the group that consumed adequate amounts of protein (1.2 g/kg/d) (Snijders et al., 2014a). However, the proportion of MuSCs expressing myostatin, an inhibitor of myogenesis (Langley et al., 2002), remained low in the protein restricted group compared to the normal protein group. A prolonged reduction in Myostatin^+^MuSCs may predict a prolonged MuSC response to training when protein is restricted; however, later time points were not examined in this study.

The general findings of the acute exercise studies suggest that protein intake in the form of whey or EAA augments the MuSC response to resistance exercise in both young and older males. Conversely, protein restriction does not impair the MuSC response to training and may prolong the response; however, future studies with longer protein restriction and later post-exercise timepoints are necessary to confirm this speculation.

#### Response to Resistance Training

Most studies suggest that resistance training increases MuSC content, primarily in type II fibres. Verdijk et al. (2014), found that 12-weeks of lower-body resistance training significantly increased type II fibre CD56^+^MuSC content in older adults. Similarly, a 12-weeks lower-body training program found that NCAM^+^MuSC content increased robustly following resistance training in extreme responders (Petrella et al., 2008). In addition, 16-weeks of lower-body resistance training in males and females aged 20–35 years increased NCAM^+^MuSC content (Petrella et al., 2006). Similarly, another 16-week study demonstrated Pax7^+^MuSC content was significantly increased in both type I and II fibres in young males (Bellamy et al., 2014). The consensus is that chronic resistance training increases MuSC content in adults of all ages.

Several resistance training studies have examined the effects of whey protein on Pax7^+^MuSC content. Some studies have shown that protein supplementation during a resistance exercise intervention did not increase MuSC content from what was seen with exercise alone (Farup et al., 2014b; Reidy P. T. et al., 2017; Snijders et al., 2018). However, Mobley et al. (2017) observed an increase in MuSC number with protein supplementation compared to placebo post-training. These inconsistent findings could be explained by the amount of protein consumed by participants in the different studies. Participants in the study detecting differences in MuSC content (Mobley et al., 2017) consumed higher (∼1.8–2.0 g/kg/d) amounts of protein compared to placebo (∼1.3 g/kg/d). However, in all studies, the placebo groups were consuming adequate amounts of protein to support muscle hypertrophy ∼1.3 g/kg/d of protein.

A particularly novel study examined the role of protein supplementation on markers of muscle regeneration during bedrest and recovery (Brooks et al., 2010). Participants were confined to bedrest for 28-days followed by 14-days of active recovery and were randomized to receive 15 g of EAA without resistance training, EAA 3-h after resistance training or 5 min before resistance training (Brooks et al., 2010). During the 14-days of active recovery, all participants completed mild endurance training, while the participants originally assigned to the resistance training group during bedrest continued their resistance training program. Only the participants that consumed the EAA supplement 3-h after resistance exercise significantly increased myonuclei per fibre, while a significant reduction in myonuclei per fibre was detected in both the group that received EAAs and remained sedentary, and the group that received EAAs 5-min before exercise compared to baseline (Brooks et al., 2010). These results suggest that the timing of amino acid delivery around exercise may be a relevant factor in the MuSC and myonuclei response.

In summary, it appears as though protein supplementation augments the increase in MuSC content following resistance exercise training compared to control diets. The limited studies in this area appear to indicate the effect of protein supplementation is dependent on dose with higher amounts (>2xRDA) and protein restriction below the RDA both potentially augmenting the MuSC response. Interestingly, the single study examining the timing of protein supplementation suggests that consuming protein a few hours after resistance exercise may provide the optimal response, at least in the context of skeletal muscle recovery following bedrest.

### The Effects of Endurance Exercise and Training With Dietary Protein Manipulation on MuSCs

Endurance exercise is the most commonly prescribed type of exercise and traditionally involves exercising at 65–70% of VO₂ _peak_ for durations of 30–60 min (Potteiger et al., 2003). Endurance exercise has historically been considered to be non- or minimally hypertrophic (Konopka and Harber, 2014). As a result, the role of MuSCs in facilitating adaptations to endurance exercise was ignored for several years. However, recently it has become appreciated that MuSCs adapt to endurance exercise by increasing their function and potentially content and play a role in non-hypertrophic muscle adaptation to endurance exercise (Joanisse et al., 2013; Nederveen et al., 2015; Abreu et al., 2017; Joanisse et al., 2018). The synergistic effects of endurance exercise paired with protein consumption are not well studied in general, and even less so in the context of MuSCs.

With respect to acute endurance exercise, Rowlands et al. (2016), examined the acute effects of different doses of protein and leucine compared to placebo on myogenic transcripts following intense endurance exercise composed of 100-min of cycling at 70–90% Wmax. In the protein/leucine supplemented condition the regenerative transcriptome was significantly upregulated compared to placebo with no difference between protein/leucine doses (Rowlands et al., 2016). With respect to endurance training, (McKenzie et al., 2016) did not find any additional beneficial effects of protein supplementation compared to placebo on MuSC content following an intervention that included 3-h of cycling for 20-days. Together, initial evidence suggests that protein supplementation augments the early myogenic response to a novel endurance exercise stimulus but does not enhance the MuSC response to endurance training.

### The Effects of Dietary Protein Manipulation and Muscle Disuse on MuSCs

Skeletal muscle atrophy can occur following a period of muscle disuse, as a consequence of disease, or in conjunction with aging (McKenna and Fry, 2017). Perturbations in MuSC activity and quantity can exacerbate skeletal muscle atrophy in various conditions (McKenna and Fry, 2017), which may be improved by protein consumption. Muyskens and colleagues (2019) demonstrated that in patients undergoing total knee arthroplasty, those who consumed 20 g of EAAs twice-daily for 7-days prior and 6 weeks following surgery had higher MuSC content on the day of surgery compared with a group consuming a placebo. Further, the group that consumed EAAs had higher *Myogenin* expression, a marker of later stage myogenesis at 1-week post-surgery, while the placebo group had higher *Myod* expression, a marker of early myogenic commitment (Ganassi et al., 2020), suggesting EAA supplementation may accelerate myogenesis (Muyskens et al., 2019). When resistance training was combined with whey protein supplementation in patients on dialysis, Molsted et al. (2015) found that the fibre-type specific increase in MuSC content was not augmented by protein supplementation compared to placebo. Lastly, in the context of aging, HMB supplementation has been shown to improve muscle recovery in rats with sarcopenia, in part by increasing MuSC proliferation (Alway et al., 2013). While in a human study, Dirks and colleagues (2017) found no additional benefits of protein supplementation compared to placebo in increasing MuSC content following resistance training in frail elderly adults. As such, protein supplementation does not appear to provide added benefits to resistance exercise in the limited research using clinical populations; however, EAAs may enhance the myogenic response following surgery.

### The Effects of Resistance Exercise and Training With Dietary Protein Manipulation on FAPs

MuSC’s contribution to muscle development, maintenance, and regeneration is regulated by a variety of cells that reside in the muscle niche. Among these cells, FAPs are an essential component of the MuSC niche, providing trophic factors that modulate MuSC activation and differentiation (Joe et al., 2010; Uezumi et al., 2010; Lukjanenko et al., 2019; Wosczyna et al., 2019). FAPs are identified by different transmembrane markers such as platelet-derived growth factor receptor alpha (PDGFRα), stem cell antigen-1 (Sca-1) in mice and PDGFRα, cluster of differentiation 90 (CD90) in humans, along with CD201, CD166, CD105, CD73 and CD15 (Joe et al., 2010; Uezumi et al., 2014a; Uezumi et al., 2014b; Arrighi et al., 2015; Uezumi et al., 2016; Kasai et al., 2017; Farup et al., 2020; Goloviznina et al., 2020; Contreras et al., 2021; Farup et al., 2021). These skeletal muscle-resident progenitors reside in the interstitial space between myofibers and sense mechanical/contractile forces (De Lisio et al., 2014). FAPs play an essential role in skeletal muscle maintenance and regeneration (Wosczyna et al., 2019; Uezumi et al., 2021); however, during aging FAPs enhance fibrotic differentiation at the expense of adipogenic differentiation (Lukjanenko et al., 2019) and many muscle diseases are characterized by enhance fibrotic differentiation of FAPs (Contreras et al., 2021; Giuliani et al., 2021). This dual role observed in FAPs during health and disease is highly determined by the muscle niche (Theret et al., 2021), of which amino acids are a crucial component, as well as their metabolism (Nguyen et al., 2019; Collao et al., 2020).

The mechanisms responsible for how exercise and protein consumption regulate FAP function is an area that warrants further investigation. In this context, Farup et al. (2015), demonstrated that 12 weeks of resistance training in humans increased of the number of proliferating PDGFRα+ and CD90+ FAPs, with no additional effect of whey protein supplementation. Interestingly, an acute muscle-damaging eccentric contraction protocol in humans showed a decrease in pericyte number, only in the protein supplemented group (De Lisio et al., 2015). Pericytes are perivascular cells in the muscle interstitium that envelop and form connections with adjacent capillary endothelial cells and line the skeletal muscle vasculature (Bergers and Song, 2005). They have some phenotypic and functional overlap with FAPs in their role in muscle regeneration and MuSC regulation (Joe et al., 2010, Birbrair et al., 2013). A recent paper by Liu et al. (2022) demonstrated transcriptomic changes occur after exercise training in old mice. Their single-cell RNA sequencing analysis indicated an increase in Igf (the gene for insulin growth factor) expression in FAPs from aged and exercise trained mice. IGF signalling is known to stimulate mTOR-Akt signalling to mediate skeletal muscle hypertrophy by promoting myogenesis and protein synthesis (Yoon, 2017, Bodine et al., 2001). These results provide a potential mechanism whereby paracrine factors, produced by FAPs could act synergistically with dietary protein to promote MPS and MuSC activation. The relationship between exercise, protein supplementation and FAP function, and the mechanisms responsible for exercise induced FAP regulation and how changes in circulating amino acid availability could impact the crosstalk between all the cells in the muscle niche, is still poorly understood.

### Perspectives and Future Directions

**FIGURE 1:**
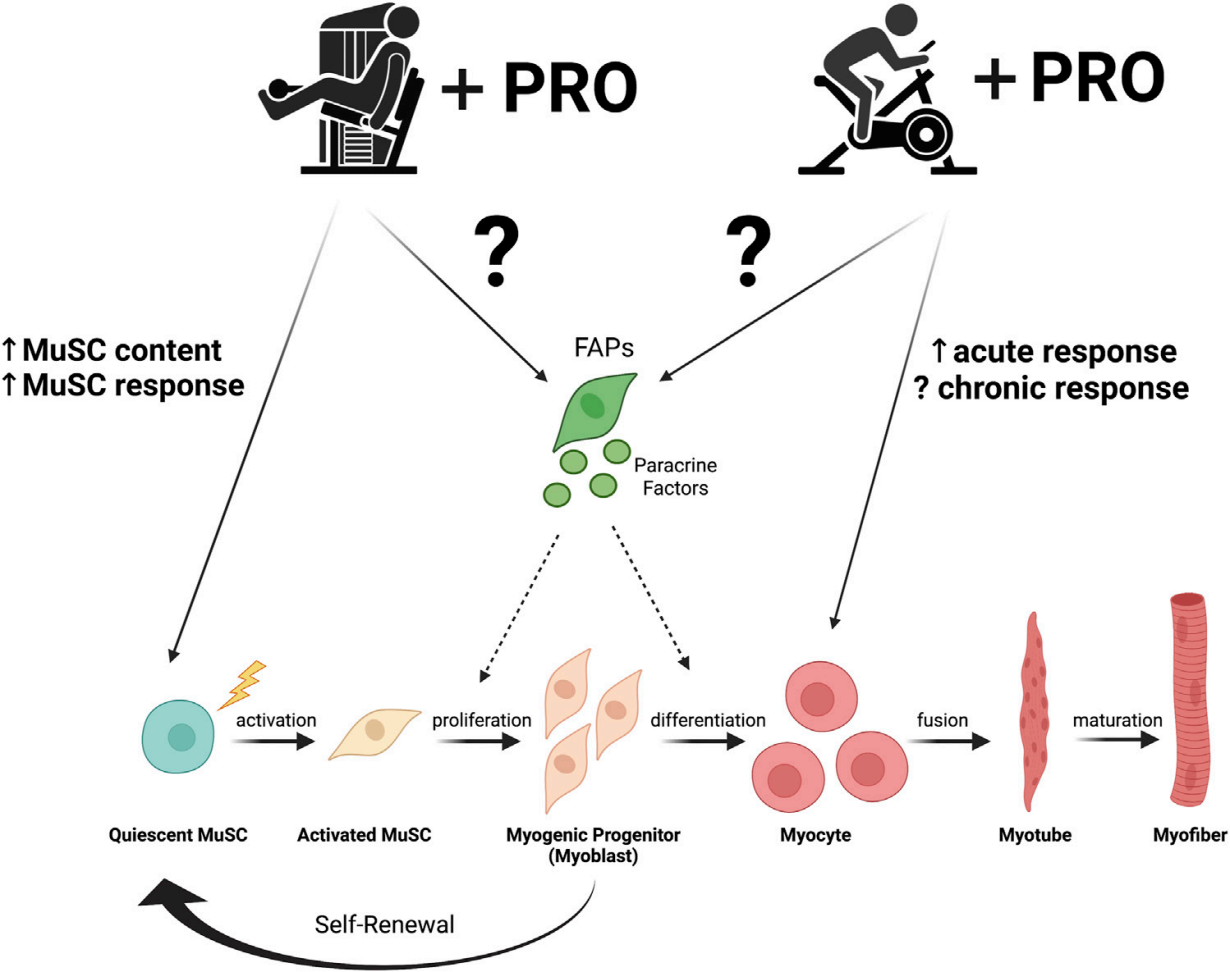
The effects of resistance and endurance exercise plus protein consumption (PRO) on muscle stem cells (MuSC) and fibro-adipogenic progenitors (FAPs). In response to skeletal muscle damage and signals from cells in their niche including FAPs, MuSCs exit from quiescence, proliferate, and differentiate to facilitate muscle repair and adaptation. Generally, findings of both acute and chronic resistance exercise suggest that protein intake augments the MuSC response in both young and older adults. Endurance exercise however is less understood. Acute exercise combined with protein consumption appears to upregulate myogenic transcripts with no effects found in chronic endurance exercise. The effects of exercise, both resistance and endurance, with protein consumption on FAP function is poorly understood and requires further investigation. Figure created with BioRender.com.

While a large body of literature has been dedicated to delineating the effects of protein supplementation on myofiber adaptations and the protein synthetic response to increased/decreased use and disease; the effects of protein and exercise on skeletal muscle cell populations, including MuSCs and FAPs have been relatively understudied. The few recent studies in this field have provided initial evidence to suggest that protein supplementation enhances the MuSC response to exercise training and that this effect may be dependent upon protein dose and timing of protein consumption relative to exercise (Figure 1). Conversely, in the context of disease, protein supplementation does not appear to provide any additional benefit to the resistance exercise induced MuSC response; however, there is a paucity of work in this area. Further, protein does not appear to enhance the FAP response to training. Given the paucity of work, the occasionally equivocal findings, and the potential for clinical application, more work is needed in this area. As with most studies in exercise physiology and skeletal muscle biology, sex-based differences should receive increased attention as most studies have exclusively or predominantly included only male participants. Further, standardized controls that are isocaloric when examining the effects of protein, or isocaloric and isonitrogenous when examining the effects of different protein sources/timing of protein ingestion should be included. The apparent discrepant response observed with respect to protein dose, with augmentation of the satellite cell response at high and low doses of protein, but no effect of moderate protein doses, requires further attention. It is interesting to speculate that interactions between protein synthesis and MuSC-mediated responses to exercise may be regulated to a certain extent by amino acid availability with sufficient amino acid availability required to maximize the protein synthetic response, while excess amino acids used to enhance the MuSC response and limited amino acids requiring an augmented MuSC response to compensate for incomplete activation of protein synthesis. Lastly, at the molecular level, determining the extent to which amino acids are directly sensed and taken up by MuSCs and other skeletal muscle cell populations via amino acid transporters could provide valuable mechanistic insights into the regulation of muscle stem/progenitor cell fate by amino acids. The field of nutritional regulation of skeletal muscle stem/progenitor cell fate is emerging alongside studies examining the effects of exercise on these same populations. Continued work examining the interaction of both diet and exercise on muscle stem/progenitor cell content and fate is expected to allow for novel and optimized therapeutic interventions for augmenting muscle function and maintaining muscle health in disease.
